# Phlpp1 Expression in Osteoblasts Plays a Modest Role in Bone Homeostasis

**DOI:** 10.1002/jbm4.10806

**Published:** 2023-08-28

**Authors:** Ismael Y Karkache, David HH Molstad, Elizabeth Vu, Eric D Jensen, Elizabeth W Bradley

**Affiliations:** ^1^ Department of Orthopedics University of Minnesota Minneapolis MN USA; ^2^ College of Veterinary Sciences University of Minnesota Minneapolis MN USA; ^3^ School of Dentistry Minneapolis MN USA; ^4^ Department of Orthopedic Surgery Stem Cell Institute, University of Minnesota Minneapolis MN USA

**Keywords:** BONE MODELING AND REMODELING, CELLS OF BONE, MOLECULAR PATHWAYS—REMODELING, OSTEOCLASTS

## Abstract

Prior work demonstrated that *Phlpp1* deficiency alters limb length and bone mass, but the cell types involved and requirement of Phlpp1 for this effect were unclear. To understand the function of *Phlpp1* within bone‐forming osteoblasts, we crossed *Phlpp1* floxed mice with mice harboring type 1 collagen (Col1a1_2.3kb_)‐Cre. Mineralization of bone marrow stromal cell cultures derived from *Phlpp1* cKO_Col1a1_ was unchanged, but levels of inflammatory genes (eg, *Ifng*, *Il6*, *Ccl8*) and receptor activator of NF‐κB ligand/osteoprotegerin (RANKL/OPG) ratios were enhanced by either Phlpp1 ablation or chemical inhibition. Micro‐computed tomography of the distal femur and L_5_ vertebral body of 12‐week‐old mice revealed no alteration in bone volume per total volume, but compromised femoral bone microarchitecture within *Phlpp1* cKO_Col1a1_ conditional knockout females. Bone histomorphometry of the proximal tibia documented no changes in osteoblast or osteoclast number per bone surface but slight reductions in osteoclast surface per bone surface. Overall, our data show that deletion of *Phlpp1* in type 1 collagen–expressing cells does not significantly alter attainment of peak bone mass of either males or females, but may enhance inflammatory gene expression and the ratio of RANKL/OPG. Future studies examining the role of *Phlpp1* within models of advanced age, inflammation, or osteocytes, as well as functional redundancy with the related *Phlpp2* isoform are warranted. © 2023 The Authors. *JBMR Plus* published by Wiley Periodicals LLC on behalf of American Society for Bone and Mineral Research.

## Graphical abstract

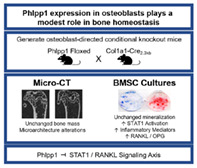

## Introduction

The skeleton renews through the process of bone remodeling, resulting in the replacement of old and/or damaged bone. Phases of bone resorption and bone formation take place during bone remodeling, with defined stages of cellular activity, including (i) activation, (ii) resorption, (iii) reversal, (iv) formation, and (v) quiescence, facilitating the bone remodeling process.^(^ ^1^, ^2^ ^)^ Bone‐resorbing osteoclasts and bone‐forming osteoblasts within the basic multicellular unit (BMU) accomplish these phases of bone remodeling.^(^ ^3^, ^4^ ^)^ Additionally, osteocytes, reversal cells, bone marrow envelop cells, and macrophages help to orchestrate this process.^(^ ^5^, ^6^, ^7^ ^)^ Disruptions to the bone remodeling process that occur because of aging, loss of reproductive status, and various skeletal pathologies lead to changes in bone mass and microarchitecture.^(^ ^8^, ^9^, ^10^ ^)^

The activities of cells within the bone‐remodeling unit are closely coordinated. Osteoblasts and osteocytes produce cytokines, including receptor activator of NF‐κB ligand (RANKL) and macrophage colony‐stimulating factor (M‐CSF), which induce osteoclastogenesis and bone resorption.^(^ ^11^ ^)^ Production of osteoprotegerin (OPG) antagonizes RANKL‐mediated osteoclastogenesis by acting as a soluble RANKL decoy receptor; thus, the ratio RANKL/OPG in part dictates levels of osteoclast differentiation.^(^ ^11^ ^)^

Phlpp1 (Pleckstrin homology [PH] domain leucine‐rich repeat protein phosphatase 1) functions as a serine/threonine protein phosphatase. Phlpp1, along with its isozyme Phlpp2, solely comprise the type 2C protein phosphatase family that function to dampen anabolic kinase signaling.^(^ ^12^ ^)^ PHLPP1 consists of several structural motifs, including Ras‐association, PH, and PDZ domains that facilitate interactions with kinases including PKC and Akt isoforms, as well as the AMKP and MAPK pathways.^(^ ^13^, ^14^ ^)^ Phlpp1 is broadly expressed but enriched within the brain and immune system.^(^ ^15^ ^)^ Prior reports demonstrate that levels of *Phlpp1* decline in iliac crest biopsies with age, but short‐term estrogen therapy restores levels.^(^ ^16^ ^)^ Traditional phosphatase inhibitors do not target the activity of PHLPP1/2, but several small molecule inhibitors (eg, NSC 117079, NSC 45586) limit their phosphatase activity and alter levels of PHLPP1/2 in vitro.^(^ ^17^ ^)^

Our prior work demonstrates a role of *Phlpp1* within the musculoskeletal system. Published work demonstrates that *Phlpp1* governs skeletal development and plays a role during musculoskeletal degeneration. Germline deletion of *Phlpp1* decreases limb and body length and leads to slight reductions in bone mass^(^ ^18^, ^19^ ^)^; however, these effects could be due to systemic changes imparted by loss of *Phlpp1* or to cell‐autonomous deficiencies within musculoskeletal cell types. In contrast, osteoclast‐directed *Phlpp1* deletion reduces bone resorption and enhances bone mass of females in an estrogen‐dependent fashion.^(^ ^20^, ^21^, ^22^ ^)^

Because of the contrasting effects of *Phlpp1* germline deletion and osteoclast‐directed ablation, we sought to characterize the role of *Phlpp1* within bone‐forming osteoblasts. Our data suggest that Phlpp1 limits STAT1 activation, resulting in enhancements to the RANKL/OPG ratio and inflammatory gene expression. Our data further suggest that osteoblast‐directed *Phlpp1* ablation does not limit attainment of peak bone mass but results in microarchitectural changes to trabecular bone.

## Materials and Methods

### Generation of *Phlpp1* null and conditional knockout mice

*Phlpp1* null (*Phlpp1* ^−/−^) mice were generated and genotyped as previously described.^(^ ^19^ ^)^ *Phlpp1* floxed (*Phlpp1* ^fl/fl^) mice^(^ ^21^ ^)^ were crossed with mice expressing Cre‐recombinase under the control of the Col1a1_2.3kb_ promoter^(^ ^23^ ^)^ to delete *Phlpp1* within type 1 collagen–expressing cells. Mice were genotyped for Cre as previously described^(^ ^24^ ^)^ for the *Phlpp1* floxed and null allele using the following primers: A: 5′‐TAGGAGAGACTAGTGACATC‐3′; B: 5′‐TGAGCTTATACGCTGTGATGC‐3′; and C: 5′‐TCAAAGTGGGAAAGGAAGGA‐3′.^(^ ^21^ ^)^ Conditional knockout animals from these crossings are referred to as Phlpp1 cKO_Col1a1_ mice and are on the C57Bl/6 background. Animals were housed in an accredited facility under a 12‐hour light/dark cycle and provided water and food *ad libitum*. All animal research was conducted according to guidelines provided by the National Institute of Health and the Institute of Laboratory Animal Resources, National Research Council. The University of Minnesota Institutional Animal Care and Use Committee approved all animal studies.

### Micro‐computed tomography of *Phlpp1* cKO_Col1a1_ mice

Right femora collected from 12‐week‐old male and female *Phlpp1* cKO_Col1a1_ mice and their control littermates were fixed in 10% neutral buffered formalin for 48 hours, wrapped in gauze, and placed in 1.5‐mL screw‐cap tubes filled with 70% ethanol. Scanning was performed using the XT H 225 micro‐computed tomography (μCT) machine (Nikon Metrology Inc., Brighton, MI, USA) set to 120 kV, 61 μA, 720 projections at two frames per projection with an integration time of 708 ms as previously described.^(^ ^25^ ^)^ Scans were performed at an isometric voxel size of 7.11 μm with a 1‐mm aluminum filter, 17 minutes per scan. Each scan volume was reconstructed using CT Pro 3D (Nikon Metrology Inc.). Reconstructions were converted to bitmap data sets using VGStudio MAX 3.2 (Volume Graphics GmbH, Heidelberg, Germany). Scans were reoriented via DataViewer (SkyScan, Bruker microCT, Kontich, Belgium) to create a new bitmap data set for consistent analysis. From these data, representative transverse slices were selected for qualitative analysis. Morphometric analysis was performed using SkyScan CT‐Analyzer (CTAn, Bruker micro‐CT, Belgium). Bruker's instructions and guidelines for analysis within the field were followed throughout analysis.^(^ ^25^ ^)^ 3D analysis of trabecular bone was performed in the distal metaphysis 0.7 mm proximal to the growth plate and extending 1.5 mm proximally toward the bone diaphysis. For the bone cortex, 2D analysis occurred in a 0.5‐mm section within the mid‐diaphysis defined as 4 mm from the growth plate. Regions of interest were set by automated contouring for the trabecular and cortical ranges, with some manual editing when necessary. Binary selection of all samples resulted in two separate global thresholds used to separate bone from surrounding tissue within the trabecular and cortical regions of interest. Parameters measured for trabecular bone include bone volume per total volume (BV/TV), bone surface per total volume (BS/TV), bone surface per bone volume (BS/BV) trabecular thickness, number, and spacing (Tb.Th, Tb.N, Tb.Sp), and connective density (Conn.D). Cortical parameters measured were BV/TV and cortical thickness (Ct.Th).

### Histology, static bone histomorphometry, and immunohistochemistry

Tibias from 12‐week‐old mice were fixed in 10% neutral buffered formalin then decalcified in 15% EDTA for 14 days. Tibias were then paraffin embedded and longitudinal 7‐μm sections were collected using the posterior cruciate ligament as a landmark for section depth. Sections were then TRAP/Fast Green stained (Sigma‐Aldrich, St. Louis, MO, USA; #387A‐1KT) or Masson's trichrome stained (Sigma‐Aldrich, #HT15‐1KT). Standardized histomorphometry was performed to assess osteoclast and osteoblast number per bone surface as well as osteoclast perimeter per bone perimeter.^(^ ^26^ ^)^ For immunohistochemical staining, we utilized the Mouse‐ and Rabbit‐specific HRP/DAB (ABC) Detection IHC Kit (Abcam, Cambridge, MA, USA; #ab64264) according to the manufacturer's specifications. Briefly, sections were deparaffinized in xylenes and a series of graded ethanol and rehydrated in water. Sections were blocked and primary directed toward PHLPP1 (EMD Millipore, Burlington, MA, USA; #07–1341, 1:50) or an irrelevant IgG control were applied and incubated overnight at 4°C. Streptavidin polyvalent secondary antibodies and biotin‐linked horseradish peroxidase (HRP) were then applied and chromogens were developed with 3,3′‐diaminobenzidine. Sections were then briefly counterstained with Fast Green, dehydrated, and coverslips were applied with resinous medium. Study staff were blinded to sample identities during analyses.

### RNA extraction and semiquantitative PCR

Total RNA was extracted from primary bone marrow stromal cell (BMSC) cultures or osteoclasts using TRIzol (Invitrogen, Carlsbad, CA, USA) and chloroform, and 1 μg was reverse transcribed using the SuperScript III first‐strand synthesis system (Invitrogen). The resulting cDNAs were used to assay gene expression via real‐time PCR using the gene‐specific primers listed in Table 1. Fold changes in gene expression for each sample were calculated using the 2^−ΔΔCq^ method relative to control after normalization of gene‐specific C_q_ values to Tubulin C_q_ values.^(^ ^21^, ^27^ ^)^ Shown are data from three females per group of each genotype with three independent replicate experiments.

**Table 1:** qPCR Primers

Gene	Forward primer sequence	Reverse primer sequence
*Phlpp1*	5′‐CTGGCGTGATAGCGGGCGAG‐3′	5′‐CCAGGCGCCGGGTAGTCTCT‐3′
*Ifng*	5′‐TTGGCTTTGCAGCTCTTCCT‐3′	5′‐GCTGATGGCCTGATTGTCTTTC‐3′
*IL‐6*	5′‐GCCCACCAAGAACGATAGTCA	5′‐ACTGGATGGAAGTCTCTTGC‐3′
*Ccl8*	5′‐TGGAAGCTGTGGTTTTCCAG	5′‐TTCAAGGCTGCAGAATTTGAGA‐3′
*OPG*	5′‐CCAAGAGCCCAGTGTTTCTT	5′‐CCAAGCCAGCCATTGTTAAT‐3′
*RANKL*	5′‐GCTGGGACCTGCAAATAAGT	5′‐TTGCACAGAAAACATTACACCTG‐3′
*Ywhaz*	5′‐GAGCTGAGCTGTCGAATGAG	5′‐GATGACCTACGGGCTCCTAC‐3′

### Western blotting

Cell lysates were collected in a buffered SDS solution (0.1% glycerol, 0.01% SDS, 0.1 m Tris, pH 6.8) on ice. Total protein concentrations were obtained with the Bio‐Rad D_C_ assay (Bio‐Rad, Hercules, CA, USA). Proteins (20 μg) were then resolved by SDS‐PAGE and transferred to a polyvinylidene difluoride membrane. Western blotting was performed with antibodies (1:2000 dilution) for phospho‐S727 STAT1 (Cell Signaling Technology, Danvers, MA, USA; #9177), STAT1 (Cell Signaling Technology, #14994), Histone 3 (Millipore, #05–928), and corresponding secondary antibodies conjugated to HRP (Cell Signaling Technology). Antibody binding was detected with the Supersignal West Femto Chemiluminescent Substrate (Pierce Biotechnology, Rockford, IL, USA). Shown are data from averaged from 3 males, but studies were performed in females (*n* = 3 per group) or males (*n* = 3 per group) of each genotype in three independent replicate experiments, each containing 12 pooled replicate wells per group.

### Bone marrow stromal cell mineralization assays

Bone marrow stromal cells were flushed from the marrow cavity of 6‐ to 8‐week‐old female *Phlpp1* ^−/−^, *Phlpp1* cKO_Col1a1_ or control littermate mice as previously described^(^ ^28^ ^)^ and cultured in osteogenic medium consisting of αMEM supplemented with 20% FBS, 50 μg/mL ascorbate, 10 mM β‐glycerol phosphate, 1 × 10^7^ M dexamethasone. Cultures were fed every 3 to 4 days with osteogenic medium. Media from *Phlpp1* ^−/−^ mice and littermate controls were preserved from feedings on days 21 to 28 and utilized in osteoclastogenesis assays. RNA was extracted, or cells were fixed and stained with Alizarin red on day 28. Three replicate experiments were performed with bone marrow cells from one mouse per sample per experiment. For experiments utilizing the PHLPP inhibitor NSC 117079 (MedChemExpress, Monmouth Junction, NJ, USA; #HY‐19819, 5 μM), BMSCs from 2 to 3 mice were pooled and cultured in osteogenic medium, and NSC 117079 was added on days 0, 7, 14, or 21, removed, and replaced with vehicle after 7 days via media change. Cultures were fixed with 10% neutral buffered formalin on day 28 or lysed for collection of RNA.

### Imaging and quantification

For BMSC experiments, images of each well were collected using a digital flatbed scanner. The average mean gray intensity for Alizarin red– and Giemsa‐stained wells was quantified using Image J software. Each experiment was repeated independently three times (*n* = 3 mice per group).

### Statistics

All study staff were blinded to sample identities during analyses of in vivo phenotypes. Data obtained are the mean ± standard deviation (SD). The *p* values were determined with the Student's *t* test when only one experimental comparison was made. For assessment of significance with greater than two conditions, we performed a one‐way analysis of variance. Specific *p* values for analyses are shown in each figure.

## Results

### *Phlpp1* ablation enhances the RANKL/OPG ratio and inflammatory mediators

Our prior work demonstrated that germline deletion of *Phlpp1* suppressed bone mass,^(^ ^18^ ^)^ but this effect could be attributed to many different factors, including roles for *Phlpp1* within growth plate cartilage during development. In contrast to this phenotype, osteoclast‐direct deletion of *Phlpp1* increased bone mass accompanied by reduced osteoclast‐mediated bone resorption within females.^(^ ^20^, ^21^, ^22^ ^)^ Because of the contrasting effects on bone mass, we sought to assess roles of *Phlpp1* within bone‐forming osteoblasts.

**Fig. 1:**
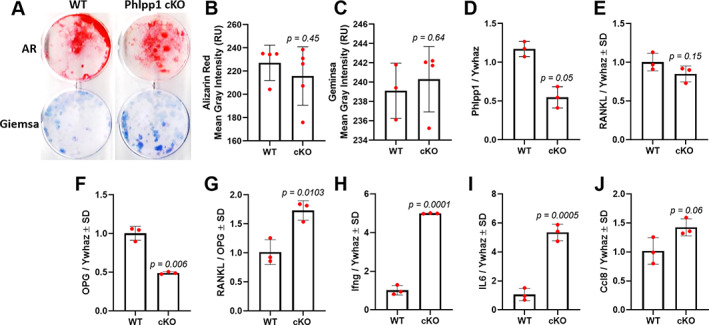
*Phlpp1* ablation enhances expression of inflammatory cytokines and the RANKL/OPG ratio. Bone marrow stromal cells from 12‐week‐old female *Phlpp1* cKO_Cola1_ mice or their control littermates were cultured in osteogenic medium for 28 days. (*A*) Alizarin red (upper wells) and Giemsa staining (lower wells) was performed. Images were scanned and the (*B*) mean gray intensity of Alizarin red–stained wells and (*C*) Giemsa‐stained wells was determined. The *p* values are as indicated. Expression of (*D*) *Phlpp1*, (*E*) RANKL, (*F*) OPG, (*G*) RANKL/OPG, (*H*) *Ifng*, (*I*) *IL‐6*, and (*J*) *Ccl8* was determined via qPCR. Specific *p* values are as indicated.

To determine cell autonomous functions of *Phlpp1* within osteoblasts, we cultured bone marrow stromal cells in osteogenic conditions but did not see changes in mineralization or CFU‐Ob (Fig. 1*A*–*D*). We confirmed that *Phlpp1* levels were diminished within *Phlpp1* cKO_Col1a1_ BMSC cultures (Fig. 1*D*). Next, we assessed expression of RANKL and OPG within these cultures. No significant change in RANKL expression was observed (Fig. 1*E*), but diminished OPG expression was evident (Fig. 1*F*), leading to a significant increase in the RANKL/OPG ratio (Fig. 1*G*). We also assessed expression of inflammatory mediators and found that *Phlpp1* cKO_Col1a1_ BMSC cultures exhibited elevated levels of *Ifng*, *IL‐6*, and *Ccl8* (Fig. 1*H*–*J*). BMSC cultures derived from *Phlpp1* germline mice also demonstrated an increased RANKL/OPG ratio and increased expression of *Ifng*, *IL‐6*, and *Ccl8* (data not shown). Although these data show that Phlpp1 may impact gene expression within osteoblasts, *Phlpp1* deletion in Col1a1‐expressing cells did not directly affect mineralization of BMSC cultures.

### Effects of Phlpp inhibition on BMSC mineralization

**Fig. 2:**
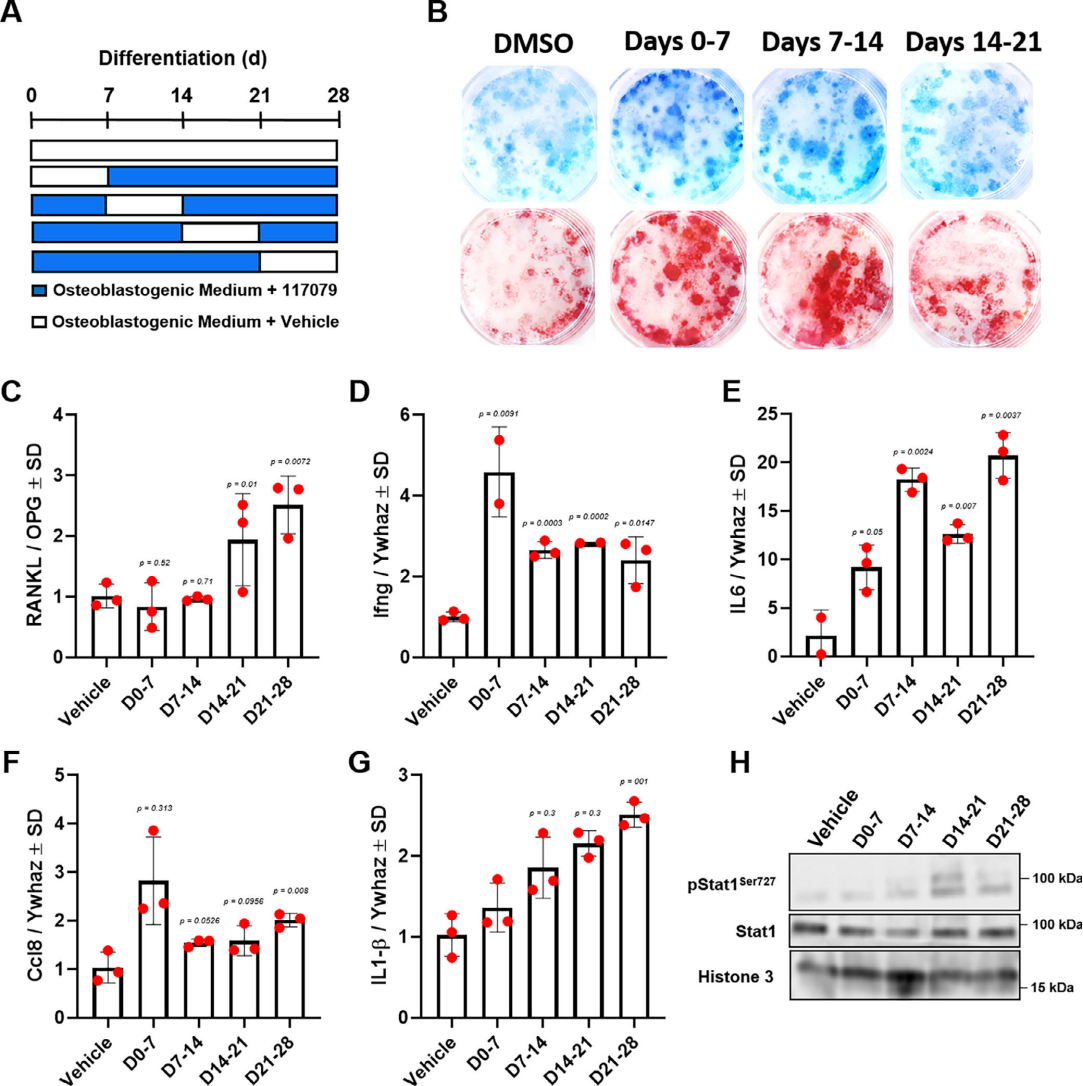
Inhibition of *Phlpp1* increases expression of inflammatory cytokines and the RANKL/OPG ratio by osteoblasts. Bone marrow stromal cells from 12‐week‐old female C57Bl6/J mice were cultured in osteogenic medium containing the Phlpp inhibitor NSC 117079 during the indicated windows of culture and DMSO at all other times for a total of 28 days. (*A*) Depiction of windowed treatment experiment. (*B*) Alizarin red (upper wells) and Geimsa staining (lower wells) was performed; duplicate wells are shown in columns. Expression of (*C*) RANKL/OPG, (*D*) *Ifng*, (*E*) *IL‐6*, (*F*) *Ccl8*, and (*G*) *IL‐1β* was determined via qPCR. (*H*) Western blotting was performed. Specific *p* values are as indicated.

To determine potential effects of the Phlpp1 small molecule inhibitor NSC 117079 on osteoblast mineralization, we collected BMSCs from 12‐week‐old C57Bl/6 female mice. BMSCs were cultured in osteogenic medium and NSC 117079 was added on days 0, 7, 14, or 21, then removed and replaced with vehicle after 7 days via media change (see depiction in Fig. 2*A*). No changes in mineralization of CFU‐Ob were observed in any condition (Fig. 2*B*). Similar to BMSC cultures derived from *Phlpp1* cKO_Col1a1_ mice, we observed an increase in the RANKL/OPG ratio when cultures were treated with the Phlpp inhibitor during days 14–21 and 21–28 (Fig. 2*C*). Enhanced expression of *Ifng*, *IL‐6*, and *Ccl8* under all conditions was also noted (Fig. 2*D–F*). Elevated levels of *IL‐1β* were also observed when cultures were treated on days 7–14, 14–21, and 21–28 (Fig. 2*G*). Enhanced phosphorylation of STAT1 accompanied these observations (Fig. 2*H*).

### Osteoblast‐directed deletion of *Phlpp1* alters bone microarchitecture

**Fig. 3:**
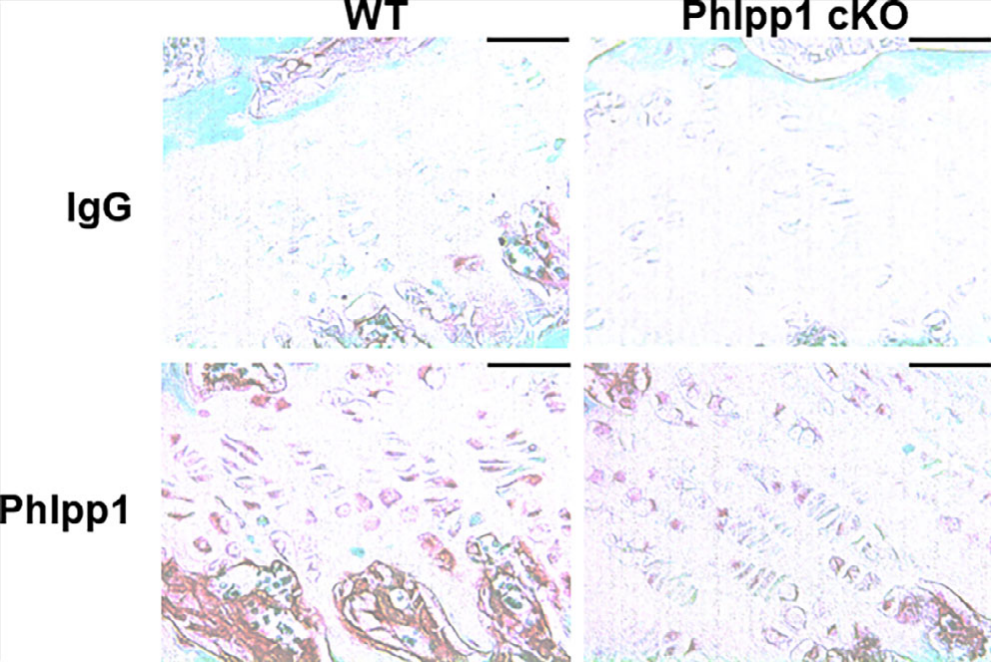
Expression of PHLPP1 by growth plate chondrocytes. Female *Phlpp1* cKO_Cola1_ mice and their control littermates were aged to 12 weeks. Tibias were decalcified, embedded, and sectioned (7 μm). Immunohistochemical staining using primary antibodies directed toward Phlpp1 or irrelevant control (IgG) was performed. Shown are images taken from the proximal growth plate (*n* = 3 per group). Scale bars = 35 μm.

**Fig. 4:**
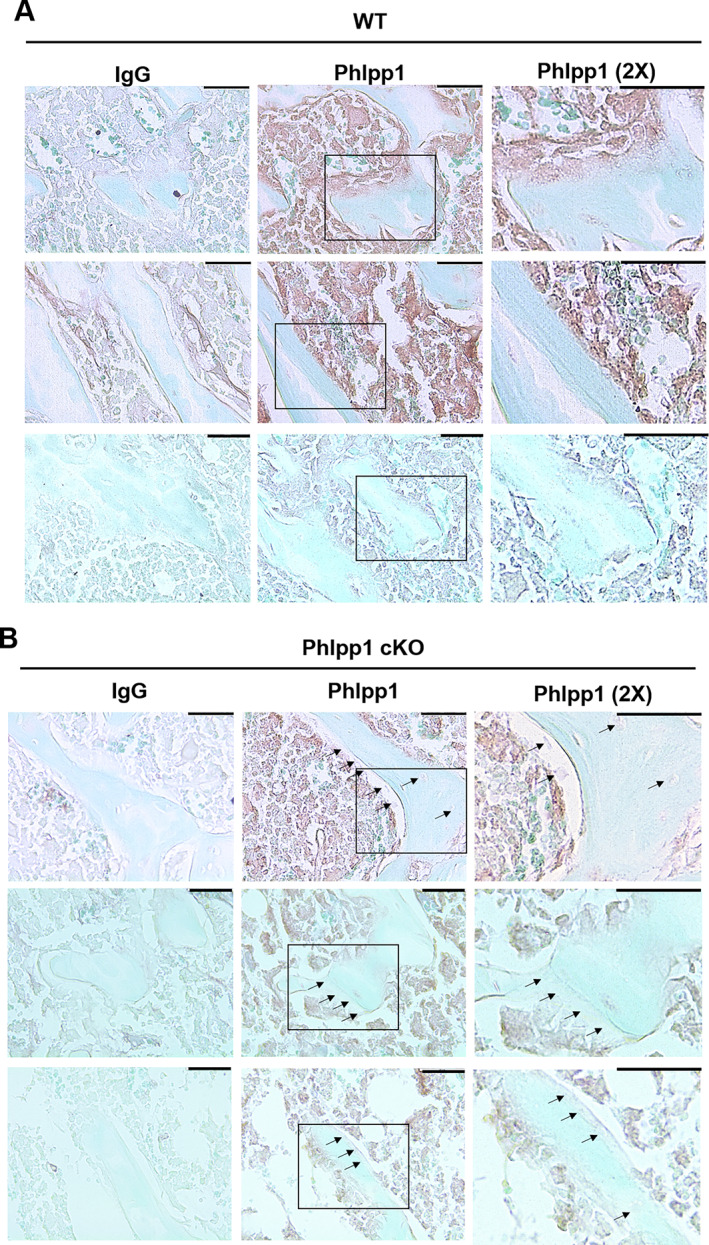
Osteoblast‐directed ablation of *Phlpp1*. Female *Phlpp1* cKO_Cola1_ mice and their control littermates were aged to 12 weeks. Tibias were decalcified, embedded, and sectioned (7 μm). Immunohistochemical staining using primary antibodies directed toward PHLPP1 or irrelevant control (IgG) was performed. Shown are images taken from the trabecular bone of (*A*) wild‐type (WT) and (*B*) *Phlpp1* cKO_Col1a1_ mice (*n* = 3 per group). Arrows note lack of positive PHLPP1 staining by bone lining cells within *Phlpp1* cKO_Cola1_ mice. Scale bars = 35 μm.

We first assessed expression of PHLPP1 within *Phlpp1* cKO_Col1a1_ mice compared with control littermates via immunohistochemistry (IHC). We confirmed PHLPP1 expression by growth plate chondrocytes as shown by prior reports^(^ ^18^, ^29^ ^)^ but did not observe loss of PHLPP1 levels within the growth plate of *Phlpp1* cKO_Col1a1_ mice (Fig. 3). In contrast, marked reductions in PHLPP1 levels occurred within bone lining osteoblasts and derived bone‐encased osteocytes compared with control littermates (Fig. 4*A*, *B*).

**Fig. 5:**
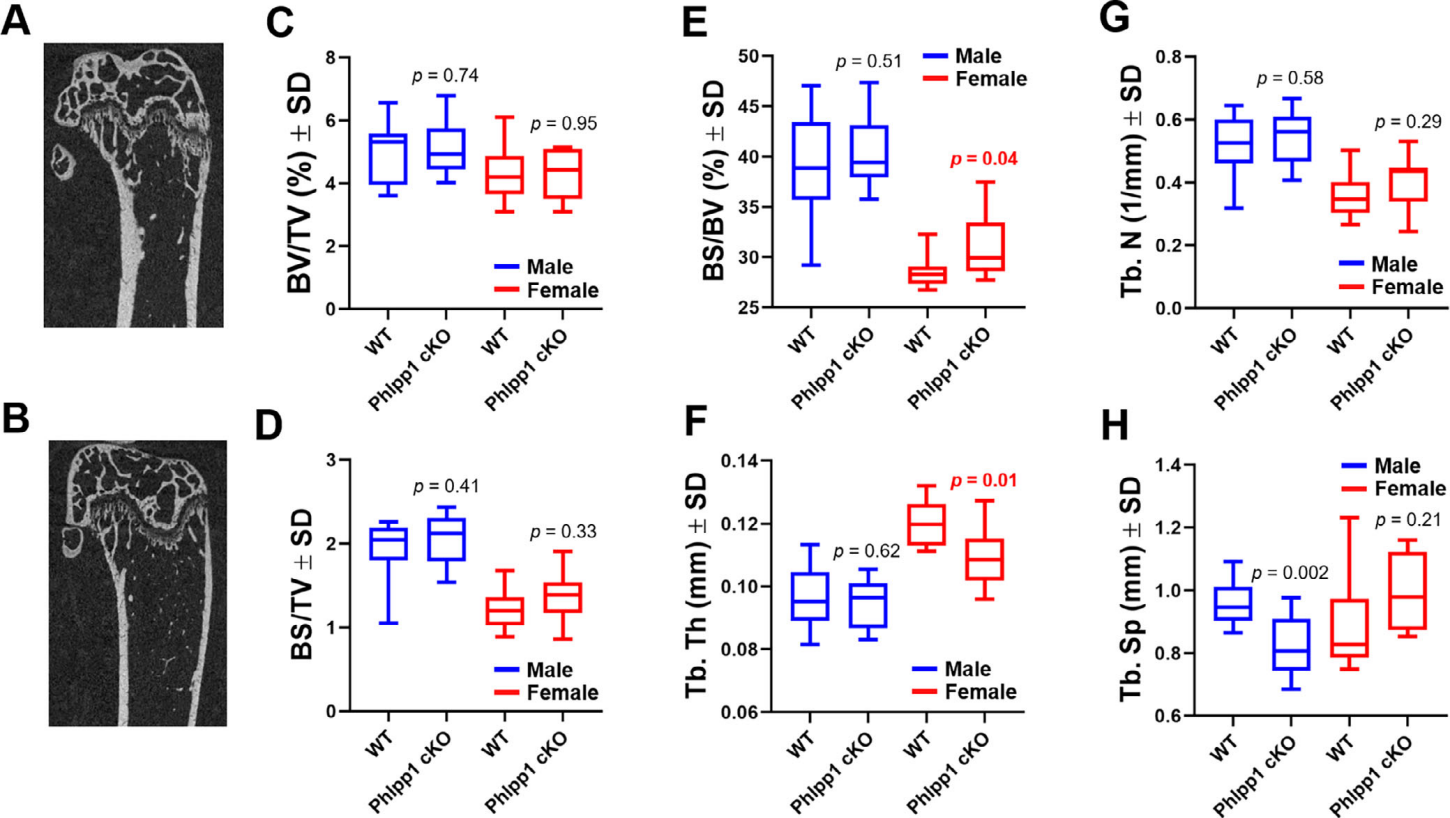
Deletion of *Phlpp1* within Col1a1‐Cre expressing cells alters bone microarchitecture. Male (*n* = 9) and female (*n* = 8) *Phlpp1* cKO_Cola1_ mice and their control littermates (*n* = 10 males and females) were aged to 12 weeks. Shown are 2D reconstructions of (*A*) control and (*B*) *Phlpp1* cKO_Col1a1_ females. Micro‐CT of the distal femora was performed, and (*C*) bone volume per total volume (BV/TV), (*D*) bone surface per total volume (BS/TV), (*E*) bone surface per bone volume (BS/BV), (*F*) trabecular thickness (Tb.Th), (*G*) trabecular number (Tb.N), and (*H*) trabecular spacing (Tb.Sp) were evaluated. The *p* values are as shown.

**Fig. 6:**
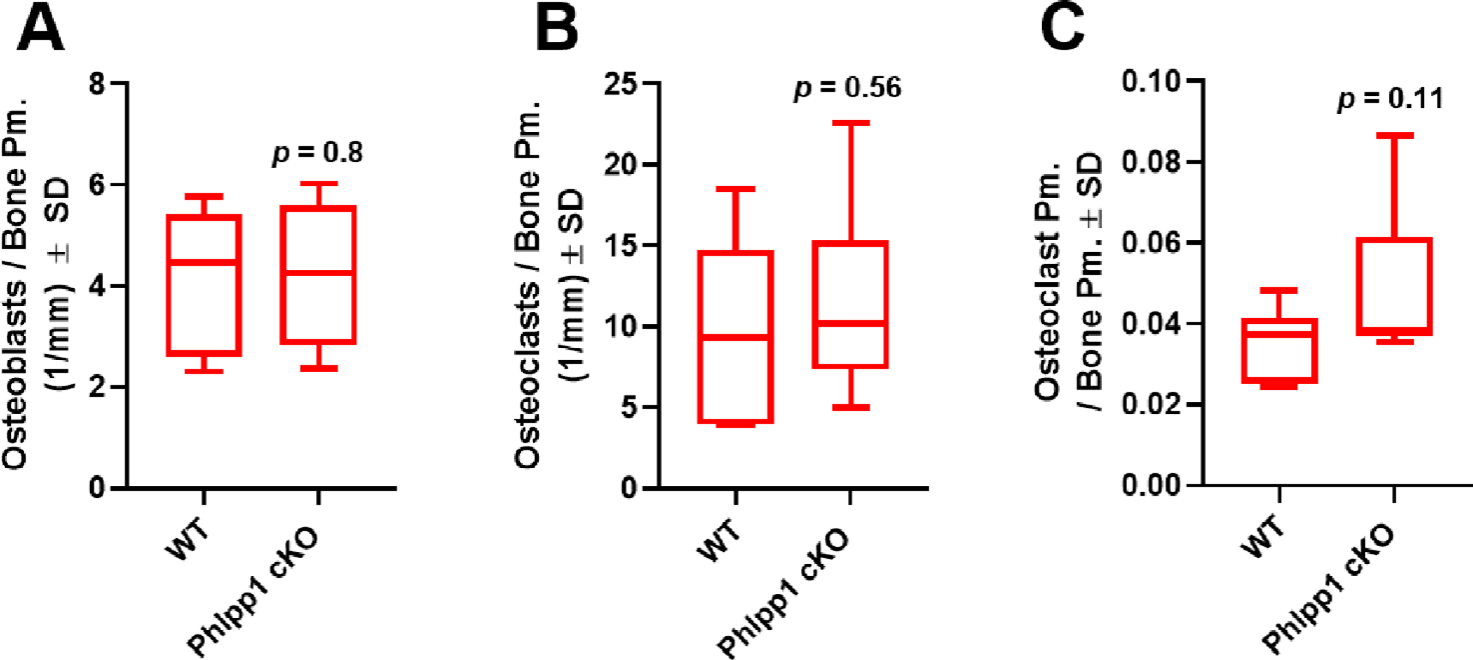
Osteoblast‐directed *Phlpp1* ablation does not impact osteoblast and osteoclast number. Female *Phlpp1* cKO_Cola1_ (*n* = 8) mice and their control littermates (*n* = 10) were aged to 12 weeks. Tibias were decalcified, embedded, and sectioned (7 μm). Standardized histomorphometry was performed to determine (*A*) osteoblasts per bone surface, (*B*) osteoclasts per bone surface, and (*C*) osteoclast surface per bone surface. The *p* values are as shown.

**Fig. 7:**
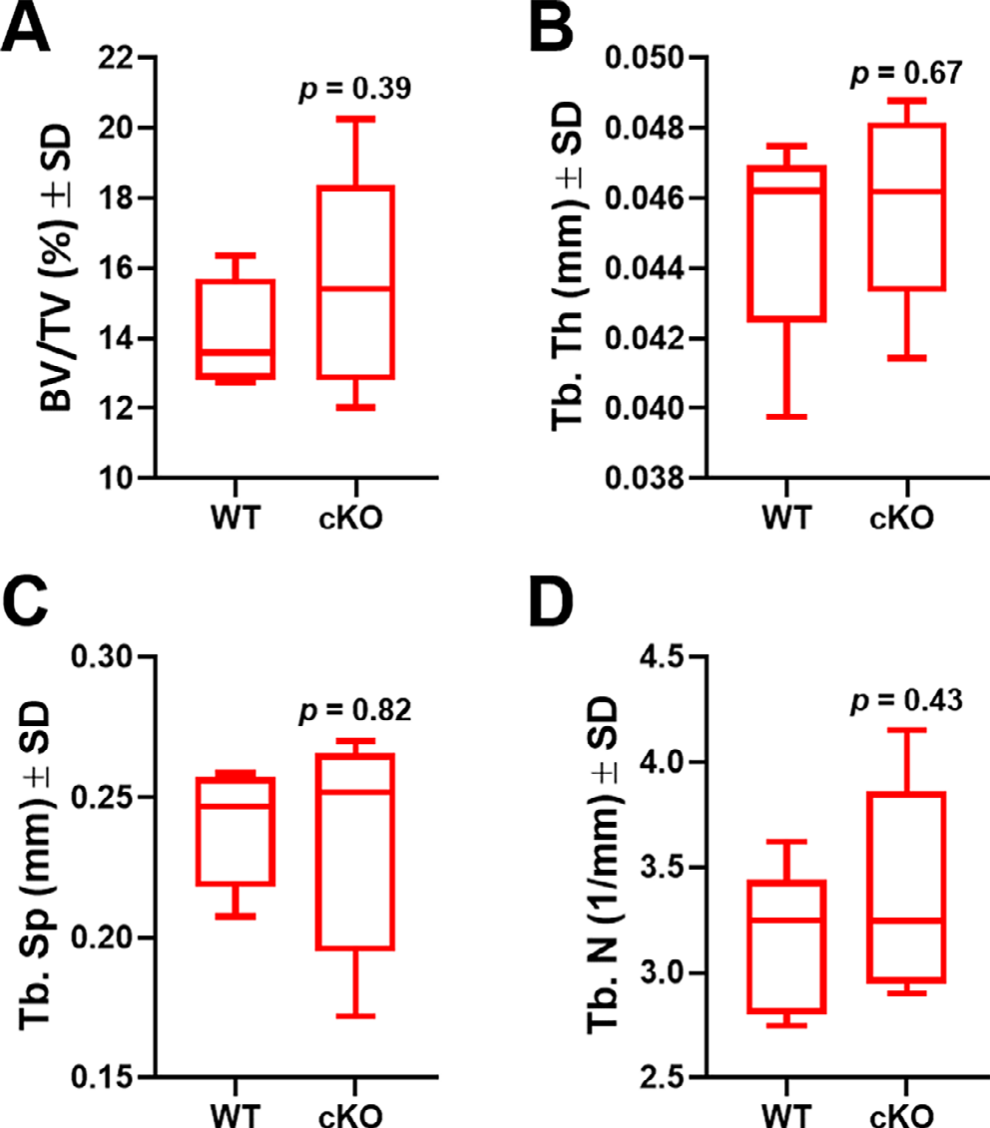
Ablation of *Phlpp1* in Col1a1‐expressing cells does not impact vertebral cancellous bone. Female *Phlpp1* cKO_Cola1_ mice and their control littermates (*n* = 5 per group) were aged to 12 weeks. Micro‐CT of the L_5_ vertebral body was performed, and (*A*) bone volume per total volume (BV/TV), (*B*) trabecular thickness (Tb.Th), (*C*) trabecular spacing (Tb.Sp), and (*D*) trabecular number (Tb.N) were measured. The *p* values are as shown.

Micro‐CT analyses of 12‐week‐old female and male *Phlpp1* cKO_Col1a1_ mice showed no significant changes in cortical thickness (data not shown), trabecular bone mass (Fig. 5*A–H*), or femoral length (data not shown) when compared with sex‐matched control littermates. Although a significant change in trabecular bone surface per total volume (Fig. 5*D*) was not evident, we did note increased cancellous bone surface per bone volume (Fig. 5*E*), indicating a potential increase in bone remodeling within female *Phlpp1* cKO_Cola1_ mice. This was accompanied by a decline in trabecular thickness (Fig. 5*F*), but no significant changes in trabecular number or spacing within *Phlpp1* cKO_Col1a1_ females (Fig. 5*G*, *H*). Trabecular spacing was diminished in *Phlpp1* cKO_Col1a1_ males (Fig. 5*H*). These data demonstrate that osteoblast‐directed deletion of *Phlpp1* compromises bone microarchitecture. We then performed standardized bone histomorphometry to assess changes in osteoblast and/or osteoclast numbers.^(^ ^26^ ^)^ Measures of osteoblasts and osteoclasts per bone perimeter were unchanged (Fig. 6*A*, *B*). We did note an enhancement of osteoclast perimeter per bone perimeter, supporting increased osteoclast size (Fig. 6*C*). Likewise, no changes in vertebral bone mass were noted (Fig. 7).

## Discussion

Our prior studies suggested a potential role for Phlpp1 during skeletal development, including attainment of peak bone mass.^(^ ^18^ ^)^ These studies were performed utilizing germline *Phlpp1* null mice, so the effects on bone mass could be attributable to developmental deficiencies we identified in growth plate chondrocyte activities,^(^ ^18^ ^)^ cell autonomous functions of skeletal cells (eg, osteoclasts, osteoblasts), or other paracrine and/or systemic effects. For instance, Phlpp1 inhibits the systemic actions of insulin and reduces survival of β cells within the pancreas, which could indirectly affect skeletal development and degeneration.^(^ ^30^, ^31^, ^32^ ^)^ Prior work also supports that *Phlpp1* ablation limits chondrocyte proliferation and endochondral ossification^(^ ^18^, ^29^ ^)^ but limits articular cartilage and intervertebral disc degeneration.^(^ ^33^, ^34^ ^)^ Likewise, Phlpp1 deficiency promotes inflammatory responses of immune cells by enhancing STAT1‐dependent signaling.^(^ ^35^, ^36^ ^)^ To refine further the functions of Phlpp1 within skeletal lineage cells, we generated mice harboring a *Phlpp1* floxed allele to allow for cell type–directed *Phlpp1* deletion.^(^ ^21^ ^)^ Our prior work demonstrated that osteoclast‐directed *Phlpp1* ablation enhanced bone mass in females by limiting osteoclast resorptive activity and facilitating the reversal process during bone remodeling.^(^ ^20^, ^21^, ^22^ ^)^

Because the phenotype observed with osteoclast‐directed *Phlpp1* ablation contrasted with that of *Phlpp1* ^−/−^ mice and the ability of Phlpp1 to attenuate anabolic kinase signaling, we sought to determine specific functions of Phlpp1 within bone‐forming osteoblasts. Unfortunately, we did not note changes in attainment of peak bone mass accompanying osteoblast‐directed *Phlpp1* deletion or effects on mineralization by bone marrow stromal cell in vitro cultures. We did note changes to expression of inflammatory cytokines, as well as enhancement of the RANKL/OPG ratio by *Phlpp1*‐deficient osteoblasts. In further support of this notion, small molecule–mediated inhibition of Phlpp1 likewise promoted expression of inflammatory cytokines and increased the RANKL/OPG ratio but did not have an effect on mineralization of BMSC cultures; thus, Phlpp1 inhibitors may not be detrimental to osteoblasts in vivo.

PHLPP1 dephosphorylates Stat1 to attenuate inflammatory signaling in macrophages.^(^ ^35^ ^)^ We likewise show that Phlpp inhibition leads to enhanced STAT1 Ser727 phosphorylation within BMSC cultures. Increased expression of inflammatory mediators, including IFN‐γ, IL‐1β, IL‐6, and Ccl8, also accompanies enhanced STAT1 activation. These inflammatory cytokines promote the recruitment and/or activity of immune cells that participate in inflammation‐induced bone loss. Prior reports demonstrate that STAT1 promotes RANKL expression by mesenchymal stromal cells to enhance osteoclastogenesis.^(^ ^37^ ^)^ Our work suggests that Phlpp1‐dependent STAT1 dephosphorylation suppresses OPG expression within BMSC cultures. Given these findings, future work examining the role of Phlpp1 in models of inflammatory bone loss, such as rheumatoid arthritis or periodontal disease, is of interest.

Lack of a phenotype associated with osteoblast‐directed ablation of *Phlpp1* in vivo could be due to several factors. PHLPP1 shares the same domain structure with its isozyme, PHLPP2, and their phosphatase domains are 68% identical.^(^ ^38^, ^39^ ^)^ Although PHLPP1and PHLPP2 do target distinct kinase isoforms (eg, Akt, PCK), their substrates do overlap considerably; thus, lack of an in vivo phenotype could be attributable to functional redundancy between Phlpp1/2 within osteoblasts. A natural future direction of this work is to assess the bone phenotype of mice with osteoblast‐directed ablation of Phlpp1 and Phlpp2 given this possibility.

Although we did not see a change in bone mass within 12‐week‐old mice, this only reflects the period of skeletal development; thus, Phlpp1 may have larger functions within osteoblast lineage cells with advancing age.^(^ ^40^ ^)^ Increased RANKL/OPG production would have the greatest effect when *Phlpp1* was removed from osteoblast‐derived osteocytes, as osteocytes are the main producers of forward RANKL signaling. It may be that 12 weeks did not provide sufficient time to ensure adequate ablation of *Phlpp1* within bone‐encased osteocytes. This may be the case as modest levels of PHLPP1 are still detected within osteocytes of *Phlpp1*‐ablated mice via IHC.

An increased RANKL/OPG ratio by *Phlpp1*‐ablated osteoblasts could also have an effect on reverse RANKL signaling within osteoblasts, leading to accelerated bone turnover rather than increased resorption in vivo.^(^ ^41^ ^)^ This is also supported by the modest increases in trabecular thickness and bone surface per bone volume detected by micro‐CT and slight increase in osteoclast surface per bone surface via histomorphometry. Although we demonstrate efficient ablation of *Phlpp1* within osteoblasts in vivo and in vitro with the Col1a12.3 kb‐Cre driver, utilization of additional osteoblast and/or skeletal mesenchymal lineage Cre drivers would help to either confirm or refute our findings.

Additionally, the lack of an effect on our in vitro mineralization assays could be attributable to the mixed population of cells within the BMSC cultures. Furthermore, the modest reduction in *Phlpp1* levels within these cultures could be reflective of other untargeted cell types within these BMSC cultures or due to incomplete deletion of *Phlpp1* within osteoblast lineage cells.

Overall, our data demonstrate that osteoblast‐directed ablation of *Phlpp1* alters bone microarchitecture and may promote bone turnover without a drastic effect on bone mass. This is accomplished through a PHLPP1‐STAT1‐RANKL signaling axis within osteoblasts.

## Author Contributions

**Ismael Y. Karkache:** Data curation; formal analysis; investigation; writing – review and editing. **David H.H. Molstad:** Data curation; formal analysis; investigation; writing – review and editing. **Elizabeth Vu:** Data curation; formal analysis; writing – review and editing. **Eric D. Jensen:** Methodology; resources; writing – review and editing. **Elizabeth W Bradley:** Conceptualization; data curation; formal analysis; funding acquisition; investigation; supervision; writing – original draft.

## Disclosures

The authors declare no conflicts of interest.

### Peer Review

The peer review history for this article is available at https://www.webofscience.com/api/gateway/wos/peer-review/10.1002/jbm4.10806.
